# Presumed prevalence analysis on suspected and highly suspected breast cancer lesions in São Paulo using BIRADS^®^ criteria

**DOI:** 10.1590/S1516-31802007000400003

**Published:** 2007-07-01

**Authors:** Vivian Milani, Suzan Menasce Goldman, Flora Finguerman, Marianne Pinotti, Celso Scazufka Ribeiro, Nitamar Abdalla, Jacob Szejnfeld

**Keywords:** Mammography, Breast neoplasm, Determination, Epidemiology, Statistics, Mamografia, Neoplasias mamárias, Detecção, Epidemiologia, Estatística

## Abstract

**CONTEXT AND OBJECTIVE:**

Breast cancer screening programs are critical for early detection of breast cancer. Early detection is essential for diagnosing, treating and possibly curing breast cancer. Since there are no data on the incidence of breast cancer, nationally or regionally in Brazil, our aim was to assess women by means of mammography, to determine the prevalence of this disease.

**DESIGN AND SETTING:**

The study protocol was designed in collaboration between the Departçment of Diagnostic Imaging (DDI), Institute of Diagnostic Imaging (IDI) and São Paulo Municipal Health Program.

**METHODS:**

A total of 139,945 Brazilian women were assessed by means of mammography beçtween April 2002 and September 2004. Using the American College of Radiology (ACR) criteria (Breast Imaging Reporting and Data System, BIRADS^®^), the prevalence of suspected and highly suspected breast lesions were determined.

**RESULTS:**

The prevalence of suspected (BIRADS^®^ 4) and highly suspected (BIRADS^®^ 5) lesions increased with age, especially after the fourth decade. Accordingly, BIRADS^®^ 4 and BIRADS^®^ 5 lesions were more prevalent in the fourth, fifth, sixth and seventh decades.

**CONCLUSION:**

The presumed prevalence of suspected and highly suspected breast cancer lesions in the population of São Paulo was 0.6% and it is similar to the prevalence of breast cancer observed in other populations.

## RESUMO

**CONTEXTO E OBJETIVO:**

Os programas de rastreamento para câncer de mama são fundamentais para a detecção precoce da doença. A detecção precoce de lesões suspeitas é essencial para diagnóstico, tratamento e possível cura do câncer de mama. Como no Brasil não há dados nacionais ou regionais sobre a prevalência do câncer de mama, nosso objetivo foi avaliar mulheres através da mamografia para determinar a prevalência da doença no Brasil no período de abril de 2002 a setembro de 2004.

**TIPO DE ESTUDO E LOCAL:**

Este trabalho foi realizado com a colaboração do Departamento de Diagnóstico por Imagem (DDI), Instituto de Diagnóstico por Imagem e da Prefeitura da cidade de São Paulo.

**MÉTODOS:**

Um total de 139.945 mulheres brasileiras foram avaliadas através de mamografias no período de abril de 2002 a setembro de 2004. Utilizando o sistema Birads^®^ ACR (Breast Imaging Reporting and Data System, American College), a prevalência de lesões suspeitas e altamente suspeitas foi determinada.

**RESULTADOS:**

A prevalência de lesões suspeitas (BIRADS^®^ 4) e altamente suspeitas (BIRADS^®^ 5) aumentou com a idade, especialmente após a quarta década. De acordo com isso, as lesões BIRADS^®^ 4 e BIRADS^®^ 5 são mais prevalentes nas quarta, quinta, sexta e sétima décadas de vida.

**CONCLUSÕES:**

A prevalência presumida de lesões suspeitas e altamente suspeitas de câncer de mama na população da cidade de São Paulo é 0,6% e esta prevalência é similar a de outras populações observadas.

## Introduction

Population-based health programs for breast cancer screening are essential for deçtecting breast tumors in their initial stages. Developed countries and the majority of developing nations have implemented breast cancer screening strategies in order to detect and treat the disease in its earliest stages. The importance of early diagnosis for treating and possibly curing breast cancer patients has been demonstrated in many studies.^1^ Disease aggressiveness and high mortality rates are considered to be related to late diagnosis.^2^

Many authors^1,3,4^ have demonstrated the importance of mammography for screening for breast cancer and achieving early diagçnosis. These studies have also indicated that early detection of breast cancer involves less cost for the health system and is associated with significantly higher cure rates, better treatment options and better responses from patients.^2^

Breast cancer is considered to be one of the most prevalent diseases worldwide. Moreover, breast cancer is the disease with the highest death rates in Western Europe and North America. In Canada and the United States, statistics have shown that one in ten women will develop breast cancer during their lifetimes.^5^ According to the Brazilian National Cancer Institute (INCA),^6^ a considerable increase in the mortality rate due to breast cancer was observed in Brazil between 1978 and 1998. About 31,500 new cases of breast cancer were estimated to have emerged in Brazil in 1999, thereby confirming the trend towards higher prevalence of the disease in developçing countries in recent years.

The most important screening proçgrams for breast cancer are based on mamçmography. This is considered to be the best method for screening for early-stage breast cancer and its use has been described as the most efficient strategy for reducing the mortality rate due to breast cancer. The sensitivity of mammography for breast cancer diagnosis ranges from 85% to 92%.^3^ Experience from screening programs for breast cancer that have been implemented around the world has suggested that mamçmography should be performed on women without symptoms every year, starting at the age of 40 years. This recommendation is supported by the American College of Radiology (ACR).^4,5,7-10^

Among the factors that may influence the development of breast cancer are the woman's age at the menarche, menopause and first pregnancy, along with late pregçnancy and parity.^4,7-9^ Other important factors involved in the development and progression of breast cancer include heredçity, genetically acquired abnormalities and other endogenous and exogenous environçmental factors.^10,11^ Five to 10% of breast cancer cases have a hereditary background and, in 3.5% of the patients, the disease is related to the presence of a genetic defect, BRCA-1, which give rise to genetic susçceptibility to breast cancer. Patients with a family history of breast cancer (mother, sister or daughter) have a two to fourfold greater chance of developing the disease.^12-14^ Among the environmental factors, radiation exposure, fat-rich diet and alcohol use may influence the development of the disease.^15^ Obesity is a risk factor for different types of tumor, including breast, prostate, colon and womb cancer.^16^

Epidemiological studies have demonçstrated that the prevalence of breast cancer according to mammographic screening surveys varies among different populations (Table 1). Depending on the population studied, breast cancer prevalence ranges from 0.40 to 0.81%.^17-22^

**Table 1.** Breast cancer prevalence in different populations around the world^17-22^

Country	Prevalence (%)	Country	Prevalence (%)
Greece	0.40	France	0.58
Spain	0.46	European Union	0.60
Portugal	0.49	USA (New York)	0.61
Italy	0.53	Finland	0.64

According to the ACR, mammography examinations should be undertaken every year by patients within the age range from 40 to 49 years, and this strategy causes a reduction of breast cancer mortality by about 19%.^7,23^ For patients under 40 years of age, mammography is suggested either when they are included in a risk group for disease development or when palpable nodules need to be investigated.^24-26^ However, despite its high sensitivity and low specificity, mammography does not detect 10 to 20% of breast cancers.^27-29^

For breast lesion classification, the Breast Imçaging Reporting and Data System (BIRADS^®^) is usually used. This standardized mammographic evaluation system was proposed by the ACR in 1992 and reviewed in 1998.^7^ The process aimed to homogenize the analysis of mammography reports and also had the objective of making the information and procedures uniform. Since 1998, the BIRADS^®^ system has been recomçmended for mammography reports in Brazil, following recommendations from the Brazilian College of Radiology and the Brazilian Federaçtion of Gynecology and Obstetrics.^6^

Widespread use of mammography as a screening tool for breast cancer diagnosis alçlows comparison of disease incidence among different population groups and brings important information for planning health prevention programs and resource allocation in developing countries. The use of standardçized mammographic analysis in accordance with the ACR recommendations has gained worldwide acceptance and motivated us to apply this method in the screening program for breast cancer in São Paulo, Brazil. Using BIRADS^®^ standard reports, in the present study we determined the presumed prevalence of suspected and highly suspected breast cancer lesions among a significant number of women living in São Paulo in 2003.

## OBJECTIVE

The aim of this study was to analyze the presumed prevalences of suspected and highly suspected breast cancer lesions in a representative sample of the population of São Paulo, Brazil, using mammographic screençing and systematic analysis by means of the ACR criteria, as standardized by BIRADS^®^.

## METHODS

### Patients

A total of 139,945 mammographic exçaminations performed between April 2002 and September 2004 were assessed retroçspectively. The study protocol was designed in collaboration between the Department of Diagnostic Imaging of Universidade Federal de São Paulo (DDI-Unifesp), the Institute of Diagnostic Imaging (IDI) and the São Paulo Municipal Health Program. All the mamçmograms came from medical facilities located in the metropolitan area of São Paulo. They were read and analyzed in the Report Center of DDI-Unifesp by radiologists who had been trained in the use of BIRADS^®^ standard reports. The study protocol had previously been submitted to and been approved by the Ethics Committee of Universidade Federal de São Paulo — Escola Paulista de Medicina (Unifesp-EPM).

### Inclusion criteria

Patients undergoing mammography examinations for breast cancer screening were included in this study. Patients with palpable nodules and patients who had alçready undergone surgery due to breast cancer were excluded.

### Mammogram technique

The conventional technique for mamçmographic screening was used, with four basic incidences: caudal-cranial and medial-lateral, bilaterally. Some complementary incidences (lateral, compressions and magnifications) were performed in approximately 15% of the mammographic screening.

### Equipment, film quality and maintenance

The examinations were performed on standard mammography equipment (GE 600T, GE 700 T, Lorad MII-E and Philips Diagnostic UC) in primary healthcare units in the São Paulo metropolitan area. The quality of the film used for mammography was supervised by radiologists specializing in mammographic radiology at all the primary healthcare units. Weekly checks were made on cleaning methods and the specific care taken with film developing and fixing. The mainteçnance procedures for the equipment were carçried out by a group of specialized technicians. Image quality control was also performed by a technical team from the Radiation Physics Department of Unifesp-EPM.

### Radiologist training

All the radiologists affiliated to the Report Center of DDI-Unifesp had had training and at least two years of experience with imaging diagnostics. All of them were familiar with the examination analysis proçtocol (the revised BIRADS^®^ system), which was followed and used as the standard for the reports.

### Lesion classification

All the lesions were classified according to the BIRADS^®^ criteria described by the ACR.^30^ These criteria are as follows: BIRADS^®^ 0 refers to the need for other mammographic examinations with other incidences (localized compression, magnification, etc) or for other methods (ultrasound). BIRADS^®^ 1 and 2 refer to negative mammograms or to typically benign lesions, respectively. Theoretically, neither of these categories have any possibility for maçlignancy, and the recommendation is annual mammographic control. BIRADS^®^ 3 refers to lesions considered to probably be benign, although there is a 2% chance of malignancy, and the recommendation is radiological control every six months. BIRADS^®^ 4 refers to lesions in which the chance of malignancy ranges from 2% to 90% (suspected lesions), and BIRADS^®^ 5 refers to lesions in which the chance of maçlignancy is greater than 90% (highly suspected lesions). In the cases of both BIRADS^®^ 4 and BIRADS^®^ 5, a biopsy is recommended to ensure an effective diagnosis.

### Statistical analysis

The presumed prevalences of BIRADS^®^ 4 and BIRADS^®^ 5 lesions in our sample were presented as numbers and percentages. The observed frequencies of lesions classified as BIçRADS^®^ 4 and BIRADS^®^ 5 were also presented according to age groups. Considering that BIçRADS^®^ 4 and BIRADS^®^ 5 lesion frequencies were homogeneous, the chi-squared test was used to compare lesion frequencies between age groups and the expected frequencies for each age group. The significance level for these frequencies was set at p < 0.05.

## RESULTS

Table 2 shows the prevalence of breast lesions according to the BIRADS^®^ classificaçtion among women living in the São Paulo metropolitan area who underwent mammoçgraphic screening. The percentages of patients who presented BIRADS^®^ 0, BIRADS^®^ 1 and BIRADS^®^ 2 in their mammograms were 11.7%, 38.1% and 49%, respectively. Lesions classified as BIRADS^®^ 3, BIRADS^®^ 4 and BIçRADS^®^ 5 were observed in 0.57%, 0.34% and 0.14% of the examinations, respectively.

**Table 2.** Mammography results from women living in the São Paulo metropolitan area according to age group and BIRADS® (Breast Imaging Reporting and Data System) classification

Age (years)	BIRADS^®^						
	0 n (%)	I n (%)	II n (%)	III n (%)	IV n (%)	V n (%)	Total n (%)
≤ **30**	535	1290	520	4	5	4	2358
	(22.7)	(54.7)	(22.0)	(0.17)	(0.21)	(0.17)	(100)
**31-40**	553	10980	7494	60	55	11	19153
	(2.8)	(57.3)	(39.1)	(0.31)	(0.29)	(0.06)	(100)
**41-50**	8390	25518	25607	272	184	50	60021
	(13.9)	(42.5)	(42.6)	(0.45)	(0.3)	(0.08)	(100)
**51-60**	4700	12129	20681	293	129	58	37990
	(12.3)	(32.0)	(54.4)	(0.77)	(0.34)	(0.15)	(100)
**61-70**	1666	2966	10291	120	71	43	15157
	(11.0)	(19.5)	(67.9)	(0.79)	(0.47)	(0.28)	(100)
**71-80**	524	467	3606	46	32	26	701
	(11.1)	(9.9)	(76.7)	(0.97)	(0.68)	(0.55)	(100)
≥ **81**	62	40	436	5	8	14	565
	(10.9)	(7.0)	(77.1)	(0.88)	(1.41)	(2.47)	(100)

Out of the total of 139,945 mammograms analyzed, most of the examinations were from women in the age groups of 41-50, 51-60 and 61-70 years (n = 134,419). Table 3 shows the frequency of breast lesions in these women who underwent mammographic screening, according to age group and BIRADS^®^ classification. The prevalence of suspected (BIRADS^®^ 4) and highly suspected (BIRADS^®^ 5) lesions increased with age, especially after the fourth decade of life.

**Table 3.** Mammography results from women living in the São Paulo metropolitan area according to BIRADS^®^ (Breast Imaging Reporting and Data System) classification

BIRADS^®^	Prevalence
0	11.7%
1	38.1%
2	49.0%
3	0.57%
4	0.34%
5	0.14%

The presumed prevalence of breast cancer in our sample is shown in Table 4. We obçserved that 11.7% of our patients were classi- fied as BIRADS^®^ 0. According to Sickles,^26^ apçproximately 10% of these lesions will require further investigation and 30% of these lesions will be considered positive, thus totaling breast cancer prevalence in this category of 0.32%. BIRADS^®^ 1 and BIRADS^®^ 2 do not represent suspected lesions and are not associated with breast cancer. On the other hand, convincçing evidence shows that 2% of BIRADS^®^ 3, 30% of BIRADS^®^ 4 and almost 100% of BIRADS^®^ 5 are positive lesions. Taking into consideration the statistical limitations, the presumed prevalence of breast cancer among the population of the greater São Paulo area was found to be around 0.6 %.

**Table 4.** Presumed prevalence of breast cancer among women living in the São Paulo metropolitan area according to BIRADS^®^ (Breast Imaging Reporting and Data System) mammographic classification

	BIRADS^®^					
	0	1	2	3	4	5
Prevalence found (%)	11.7	38.1	49.0	0.57	0.34	0.14
Presumed cancer prevalence per BIRADS^®^ category (%)	0.3	0	0	2	30	100
Total presumed cancer prevalence (%)	0.35	0	0	0.014	0.10	0.14
Total presumed cancer prevalence (%)				0.6		

## DISCUSSION

In this study, the presumed prevalence of breast cancer was determined among a signifiçcant sample of the Brazilian population, living in the greater São Paulo area. For this, mamçmographic exams were used in conjunction with controlled and uniform analysis.

Concerning the presumed prevalence of breast cancer in different BIRADS^®^ categories, it was seen that 11.7% of the patients were characterized as BIRADS^®^ 0 (Table 3). The parameters established by Sickles^26^ were taken into consideration. According to these paçrameters, approximately 10% of these lesions should undergo further investigation and 30% of these same lesions will probably be considçered positive, thus totaling 0.32%. BIRADS^®^ 1 and BIRADS^®^ 2 do not present suspected lesions. It can be taken as reasonably certain that 2% of BIRADS^®^ 3, 30% of BIRADS^®^ 4 and almost 100% of BIRADS^®^ 5 will be considered positive. Thus, the presumed prevalence of breast cancer in this sample of the population of São Paulo was 0.6%.

When this presumed prevalence is comçpared with what is observed worldwide, it can be seen that this Brazilian finding is apparently very similar to results from New York, greater than findings from Greece, Spain, Portugal, Italy, France and the European Union, and smaller than findings from Finland, Germany, Ireland, Great Britain, Luxembourg, Sweden, Denmark, Belgium and Holland (Table 1).

Brazil does not provide an official proçgram for breast cancer screening. One of the drawbacks of not adopting this practice as the routine is the cost of mammographic screençing. Nonetheless, informal screening takes place on the basis of medical advice. In the countries with high incidence of breast cancer, patients over 40 years of age are required to undergo mammographic screening. On the other hand, the recommendations for mamçmographic screening should be based on the benefits to these patients, without considering political or economic issues that could change the follow-up protocols. However, under any circumstances, the cost of treating breast tumors that are detected through screening is considerably lower than the cost when they are detected through clinical procedures.^6^

Because Brazil does not provide official mammography screening programs, with adequate data collection, there are no national statistics for the prevalence of breast cancer diçagnosed by imaging exams. Until the year 2002, all the mammography reports in the public secçtor clinic were hand-written, and there was no databank with the results. This made it difficult to recover epidemiological data. The sample that was obtained in this study came from outpatient examinations carried out at primary healthcare units in a downtown area of São Paulo.

To have the assurance that mammoçgraphic screening is able to reduce breast cancer mortality, the examination must present high sensitivity and adequate imagçing and there must be quality control within established patterns. It is well known that badly performed examinations generate falseçnegative results^16^ and can cause great harm because of this inadequate screening and the subsequent lack of treatment. The work performed at the DII gave special attention to quality control, to ensure that the women benefited from the program.

Many factors can influence mammography readings and lead to wrong interpretations, as well as to false-negative and false-positive results. Among these factors are the positioning of the breast in the mammography machine, the quality of the image, the equipment used and the radiologist's experience.^23^ In the priçmary healthcare units that had partnerships with DII, all the equipment (mammography machines, processors and negatoscopes) underçgoes periodic evaluation, focusing on checking its technical condition, in accordance with the Ministry of Health specifications, thereby asçsuring optimal working conditions not only for doctors but also for technicians.

According to the literature, the number of examinations that require supplementation (BIRADS^®^ 0) is estimated to be 10%.^30^ Bird et al. analyzed 215,665 mammographic screençings and found a recall rate ranging from 1.9% to 13.4%. The ACR considers that a rate of less than 10% of the all of the examinations is satisfactory.^31^

The ACR standards include medical auçditing of mammographic screening, and this is the best method for evaluating the ability to detect breast cancer. The primary value of such auditing consists of self-assessment of the mammography service, with the aim of revealçing the strengths and weaknesses of radiologists’ practices. In the IDI Report Center, auditing is performed by two experienced radiologists on approximately 10% of the examinations, thus ensuring the quality of the work.^3^

The partnership between DII and the São Paulo municipal authorities has emphasized good quality work and its program was able to bring together a significant volume of mammography screening samples in less than a year. These were read by a homogeneous medical team with adequate training, using the BIRADS^®^ classification and thereby standçardizing the results. Through organizing this Report Center to assist the city of São Paulo, we were able to obtain uniform information.

With this objective and organized infraçstructure, it was possible to implement this program. The aim now is to motivate other Brazilian cities and states to follow suit. Screening is not the solution for breast cancer problems, but it is a strategy that is available for detecting this disease in its initial stages, thus allowing effective and early treatment, in an attempt to decrease the cost to these patients.

## CONCLUSION

The presumed prevalence of suspected and highly suspected breast cancer lesions in the population of São Paulo was 0.6%, from studying mammograms from a screened population of 139,945 women, at the IDI Report Center in collaboration with DDI, Unifesp-EPM.

The presumed prevalence found in this study was comparable to what has been found in other populations around the world for whom mammographic screening programs have been implemented.
